# Age-Related Effects of AT1 Receptor Antagonist Losartan on Cognitive Decline in Spontaneously Hypertensive Rats

**DOI:** 10.3390/ijms25137340

**Published:** 2024-07-04

**Authors:** Jana Tchekalarova, Petja Ivanova, Desislava Krushovlieva

**Affiliations:** 1 Department of Behavioral Neurobiology, Institute of Neurobiology, Bulgarian Academy of Sciences, 1113 Sofia, Bulgaria; ivanova.petya91@gmail.com (P.I.); daisyveko@gmail.com (D.K.); 2 Department of Organic Chemistry, University of Chemical Technology and Metallurgy, 1756 Sofia, Bulgaria

**Keywords:** losartan, aging, Aβ_1–42_, CREB, oxidative stress, rat

## Abstract

Both hypertension and aging are known to increase the vulnerability of the brain to neurovascular damage, resulting in cognitive impairment. The present study investigated the efficacy of the antihypertensive drug losartan on age- and hypertension-associated cognitive decline and the possible mechanism underlying its effect in spontaneously hypertensive rats (SHRs). Losartan was administered (10 mg/kg, i.p. for 19 days) to 3- and 14-month-old SHRs. Age-matched Wistar rats were used as controls. Working memory, short-term object recognition, and spatial memory were assessed using the Y-maze, object recognition test (ORT) and radial arm maze (RAM) test. The expression of markers associated with aging, oxidative stress, and memory-related signaling was assessed in the frontal cortex (FC) and hippocampus. Motor activity measured over 24 h was not different between groups. Middle-aged vehicle-treated SHRs showed poorer performance in spontaneous alternation behavior (SAB) and activity in the first Y-maze test than their younger counterparts, suggesting age-related reduced “decision making” and reactivity in a novel environment. Losartan improved the age- and hypertension-induced decline in short-term recognition and spatial memory measured in the ORT and the second Y-maze test, particularly in the middle-aged rats, but was ineffective in the young adult rats. Changes in memory and age-related markers such as cAMP response element-binding protein (CREB) and amyloid-β_1–42_ (Aβ_1–42_) and increased oxidative stress were observed in the hippocampus but not in the FC between young adult and middle-aged vehicle-treated SHRs. Losartan increased CREB expression while reducing Aβ_1–42_ levels and concomitant oxidative stress in middle-aged SHRs compared with vehicle-treated SHRs. In conclusion, our study highlights the complex interplay between hypertension, aging, and cognitive impairment. It suggests that there is a critical time window for therapeutic intervention with angiotensin II type 1 receptor blockers.

## 1. Introduction

Hypertension, or high blood pressure, is a known risk factor for several cardiovascular and cerebrovascular diseases. The brain is particularly vulnerable to the effects of hypertension, especially the hippocampus, a critical region for memory and learning [1]. High blood pressure can affect the brain’s blood vessels, leading to conditions such as cerebral small vessel disease. This can lead to reduced blood flow, microbleeds, and other vascular changes. Hypertension can also cause structural changes in the hippocampus, such as reduced synaptic density, changes in dendritic morphology, and blood vessel damage [2]. In addition, chronic hypertension is associated with neuroinflammation and increased oxidative stress in the brain, which may induce various neurodegenerative processes, including those that are detrimental to memory. Therefore, pathological changes resulting from untreated hypertension may contribute to accelerated aging and subsequent cognitive decline. Spontaneously hypertensive rats (SHRs) are derived from Wistar Kyoto (WKY) rats, which are used as controls in hypertension studies [3]. They have a genetic predisposition to hypertension, making them a valuable model for studying the genetic and epigenetic factors that contribute to hypertension. SHRs are often more active and show higher levels of locomotor activity when placed in novel environments compared to WKY rats [4]. The progressive increase in arterial blood pressure over time in the SHRs, along with the development of brain atrophy, neuronal loss, and glial responses, resembles the pathological processes observed in the human hypertensive brain [5]. This similarity makes SHRs a valuable tool for investigating the mechanisms underlying hypertensive brain damage and for testing therapeutic interventions aimed at mitigating or preventing these detrimental effects. Studies in SHRs, including our recent report, have shown that hypertension-induced deleterious changes in the brain of SHRs, including poorer performance on memory tasks compared with normotensive rats, are evident as early as young adult rats [6,7,8]. Several groups have reported that older SHRs exhibit more severe cognitive impairment compared with WKY rats of the same age, accompanied by increased levels of apoptotic cells and oxidative stress in the hippocampus compared with normotensive rats, suggesting that oxidative stress may play a role in hypertension-associated brain damage [9,10]. The presence of apoptotic cells in the hippocampus underscores the potential impact of hypertension on cognitive function [10]. In addition, the association with oxidative stress highlights a possible mechanism underlying hypertension-related brain damage, further emphasizing the importance of investigating oxidative stress pathways in the context of hypertension and cognitive decline. While the normotensive WKY rats experienced health complications and a risk of death after 18 months, the SHRs showed a temporal deterioration in cardiovascular function and an increased susceptibility to spontaneous death at 15 months of age [11,12]. Furthermore, experimental ischemia–reperfusion studies confirmed that acute brain injury was more severe in middle-aged SHRs compared with young adult SHRs, suggesting that SHRs are susceptible to cognitive decline, neurodegeneration, and hippocampal damage that are exacerbated by aging [13]. These findings highlight the importance of understanding the mechanisms underlying these processes and exploring the window of opportunity for potential interventions to mitigate brain damage in hypertensive rats.

Losartan belongs to a class of antihypertensive drugs known as angiotensin II receptor blockers (ARBs). Angiotensin II has been shown to contribute to oxidative stress and inflammation in the brain [14], which can impair cognitive function [15]. By blocking angiotensin II receptors, losartan may attenuate these effects [16]. Recently, we reported that Wistar rats are a more appropriate control group than WKY rats for assessing cognitive impairment in young adult SHRs [8]. Hypertensive rats showed impairments in spatial working memory and short-term associative memory compared with Wistar rats but there were no significant differences in these memory aspects between SHRs and WKY rats. WKY rats showed lower levels of spontaneous motor activity compared to Wistar rats. This hypoactivity may potentially affect their performance in memory tests, such as the Y-maze test, object recognition test (ORT), and radial arm maze (RAM) test. The differences in cognitive function between SHRs and Wistar rats may be related to alterations in the hippocampal brain-derived neurotrophic factor (BDNF) signaling pathway, which is closely involved in functions related to neuronal survival, synaptic plasticity, and thus, cognition, particularly in the hippocampus. In the present study, we investigated the efficacy of losartan in ameliorating hypertension-induced memory impairment in young adult and middle-aged SHRs. We report that long-term treatment with the antihypertensive drug losartan was able to attenuate memory decline and associated biochemical changes in the hippocampus in middle-aged rats but was ineffective in young adult rats, suggesting a specific time window for therapeutic efficacy of this angiotensin II type 1 receptor blocker (ARB) against hypertension-associated cognitive decline. The underlined mechanism of the drug’s effects on cognitive functions under hypertensive conditions in middle-aged SHRs is discussed.

## 2. Results

**Figure 1:**
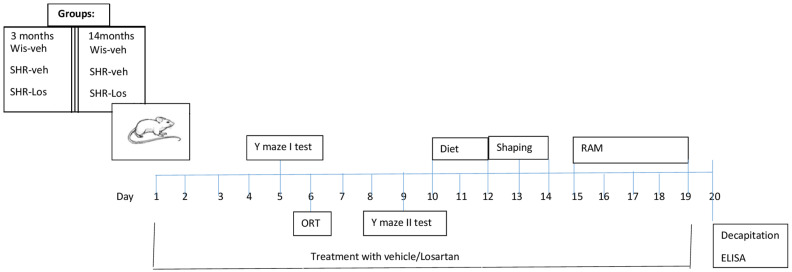
Timeline of experimental steps. Abbreviations: Wistar (Wis), vehicle (veh), spontaneously hypertensive rat (SHR), losartan (Los), object recognition test (ORT).

The experimental timeline is shown in Figure 1.

### 2.1. Aging and Losartan Treatment Do Not Affect Motor Activity in Spontaneously Hypertensive Rats, Measured over 24 h with an Actimeter

The chronobiological characteristics of SHRs have been previously studied and compared with WKY rats regarding the diurnal rhythms of several physiological parameters, including heart rate, respiration, and blood pressure [17,18]. It has been reported that aged SHRs have disturbed diurnal rhythms of respiration and blood pressure compared with WKY rats [17]. In the present study, we investigated the effect of age and hypertension on diurnal oscillations of motor activity by comparing young adult and middle-aged Wistar rats and SHRs. The possible effect of losartan treatment in 3- and 14-month-old SHRs was also investigated. Each rat was placed in the actimeter apparatus to measure its motor activity (distance and locomotor activity) for twenty-four hours. Movement detected by the tracking device was displayed as counts. 

**Figure 2:**
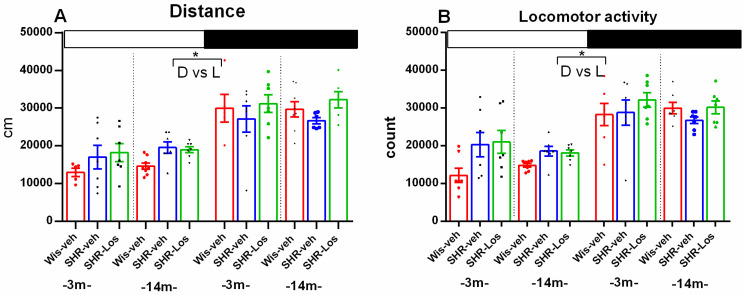
Effect of 3-month-old vehicle-treated Wistar rats (Wis-veh), vehicle-treated SHRs (SHR-veh), and losartan-treated SHRs (SHR-Los); and 14-month-old vehicle-treated Wistar rats (Wis-veh), vehicle-treated SHRs (SHR-veh), and losartan-treated SHRs (SHR-Los) on diurnal changes in distance (cm) (**A**) and locomotor activity (counts) (**B**), measured in the actimeter for 24 h. Light and dark phases are indicated by open and black rectangles above the columns. Data are presented as mean ± SEM, n = 7. Three-way ANOVA showed a main phase effect for distance [F1,55 = 55.287, *p* < 0.001] and for locomotor activity [F1,55 = 49.172, *p* < 0.001]. Post hoc Bonferroni test: * *p* < 0.05, dark phase vs. light phase within a group (**A**,**B**).

As nocturnal rats, vehicle-treated Wistar control rats aged 3- and 14-months (Wis-veh 3m and Wis-veh 14m) as well as vehicle-treated SHRs (SHR-veh 3m and SHR-veh 14m) and losartan-treated SHRs (SHR-Los 3m and SHR-Los 14m) showed a daily pattern of distance and locomotor activity (*p* < 0.05, dark phase vs. light phase within a group) (Figure 2A,B).

There was no significant difference in distance travelled and locomotor activity during the light and the dark phases between Wistar rats and vehicle-treated SHRs of the same age (young adult and middle-aged), nor between vehicle-treated SHRs and losartan-treated SHRs of the same age (*p* > 0.05). 

### 2.2. Losartan Treatment Had a Significant Beneficial Effect on Age-Related Memory Decline in SHRs

**Figure 3:**
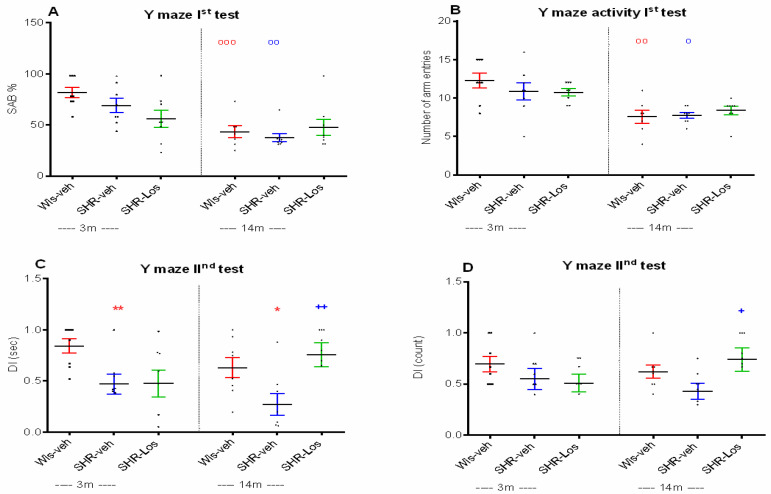
Effect of 3-month-old vehicle-treated Wistar rats (Wis-veh), vehicle-treated SHRs (SHR-veh), and losartan-treated SHRs (SHR-Los); and 14-month-old vehicle-treated Wistar rats (Wis-veh), vehicle-treated SHRs (SHR-veh), and losartan-treated SHRs (SHR-Los) on spontaneous activity behavior (SAB) (**A**), number of arm entries (**B**) in the first Y-maze test, discrimination index (DI) (sec) (**C**), and DI (counts) (**D**) in the second Y-maze test. Data are presented as mean ± SEM, (n = 8). Two-way ANOVA showed a main effect of age: [F1,31 = 39.51, *p* < 0.001] (**A**); [F1,31 = 19.895, *p* < 0.001] (**B**); a strain effect: [F1,31 = 7.247, *p* = 0.012], a treatment effect: [F1,31 = 6.38, *p* = 0.017], an age x treatment effect: [F1,31 = 6.174, *p* = 0.019] (**C**); a treatment effect: [F1,31 = 4.287, *p* = 0.048]. Post hoc Bonferroni test: ^ooo^ *p* < 0.001, Wis-veh 14m vs. Wis-veh 3m; ^oo^ *p* < 0.01, SHR-veh 14m vs. SHR-veh 3m (**A**); ^oo^ *p* < 0.01, Wis-veh 14m vs. Wis-veh 3m; ^o^ *p* < 0.05, SHR-veh 14m vs. SHR-veh 3m (**B**); ** *p* < 0.01, SHR-veh 3m vs. Wis-veh 3m; * *p* < 0.05, SHR-veh 14m vs. Wis-veh 14m; ^++^ *p* < 0.01, SHR-Los 14m vs. SHR-veh 14m (**C**); ^+^ *p* < 0.05, SHR-Los 14m vs. SHR-veh 14m (**D**). The color of the symbols corresponds to the color of the group with a significant difference.

The Y-maze apparatus was set up with three steel arms positioned at 120° to each other. In the first Y-maze test, spontaneous alternation behavior (SAB) (working memory) was assessed by allowing the rat to explore all three arms without successfully entering the same arm. Both middle-aged vehicle-treated control Wistar rats and vehicle-treated SHRs had reduced SAB and activity in the first Y-maze test compared to young adult matched vehicle-treated rats (*p* < 0.001, Wis-veh 14m vs. Wis-veh 3m; *p* < 0.01, SHR-veh 14m vs. SHR-veh 3m) (Figure 3A); (*p* < 0.01, Wis-veh 14m vs. Wis-veh 3m; *p* < 0.05, SHR-veh 14m vs. SHR-veh 3m) (Figure 3B). 

The second Y-maze test, performed at least 5 days after the first one, consisted of a pretest and a test. During the pretest, one of the arms was closed, restricting the tested rat to freely explore only two arms. The rat was individually placed in the arm (A) and had free access to two arms of the maze, labeled A and B, for a 10 min test. After 30 min, the third arm (C) was opened, allowing the animal to explore all three arms for a 5 min test freely. The 3- and 14-month-old SHRs showed impaired short-term memory compared to the age-matched vehicle-treated Wistar controls (*p* < 0.01 and *p* < 0.05, respectively) (Figure 3C). 

While losartan administration did not affect SAB in either age group of SHRs (Figure 3A,B), it significantly increased the discrimination index (DI) of 14-month-old SHRs in the second Y-maze test (*p* < 0.01 and *p* < 0.05, SHR-Los 14m vs. SHR-veh 14m) (Figure 3C,D).

The object recognition test (ORT) is a highly validated test based on the tendency of rodents to interact more with a novel than with a familiar object. It is most commonly used to assess the plausible enhancing effects of drugs on recognition memory or the effects of genetics or age on short-term memory.

**Figure 4:**
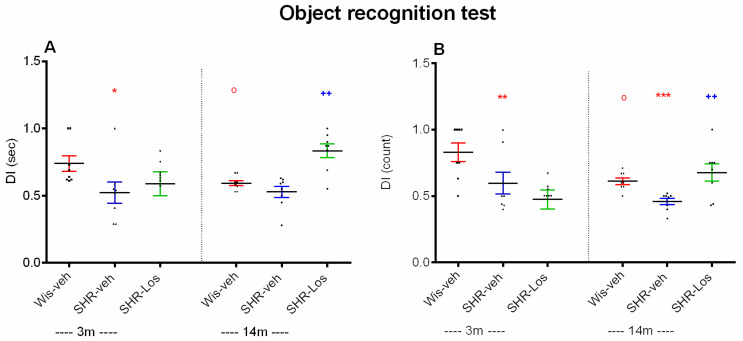
Effect of 3-month-old vehicle-treated Wistar rats (Wis-veh), vehicle-treated SHRs (SHR-veh), and losartan-treated SHRs (SHR-Los); and 14-month-old vehicle-treated Wistar rats (Wis-veh), vehicle-treated SHRs (SHR-veh), and losartan-treated SHRs (SHR-Los) on DI (sec) (**A**) and counts (**B**) measured in the object recognition test. Data are presented as mean ± SEM, n = 8. Two-way ANOVA showed a main effect of age: [F1,31 = 7.391, *p* = 0.011], strain: [F1,31 = 6.659, *p* = 0.015], age × treatment interaction: [F1,31 = 7.391, *p* = 0.011] (**A**); age effect: [F1,31 = 9.993, *p* = 0.004], strain [F1,31 = 11.595, *p* = 0.002], and age × treatment interaction: [F1,31 = 7.094, *p* = 0.013] (**B**). Post hoc Bonferroni test: ^o^ *p* < 0.05 Wis-veh 14m vs. Wis-veh 3m; * *p* < 0.05, SHR-veh 3m vs. Wis-veh 3m; ^++^ *p* < 0.01, SHR-Los 14m vs. SHR-veh 14m (**A**); ^o^ *p* < 0.05, Wis-veh 14m vs. Wis-veh 3m; ** *p* < 0.01, SHR-veh 3m vs. Wis-veh 3m; *** *p* < 0.001, SHR-veh 14m vs. Wis-veh 14m; ^++^ *p* < 0.01, SHR-Los 14m vs. SHR-veh 14m (**B**). The color of the symbols corresponds to the color of the group with a significant difference.

While the middle-aged vehicle-treated Wistar rats showed impaired recognition memory compared to the young adult rats (*p* < 0.05), there was no age-related difference in the vehicle-treated SHRs (*p* > 0.05) (Figure 4A,B). Furthermore, young adult and middle-aged vehicle-treated SHRs exhibited memory decline compared with the age-matched groups of control Wistar rats ((*p* < 0.05, SHR-veh 3m vs. Wis-veh 3m) (Figure 4A) and (*p* < 0.01, SHR-veh 3m vs. Wis-veh 3m; *p* < 0.001, SHR-veh 14m vs. Wis-veh 14m) (Figure 4B)).

Losartan treatment improved short-term recognition memory in SHRs rats, which was evident in middle-aged rats (*p* < 0.01, SHR-Los 14m vs. SHR-veh 14m) (Figure 4A,B).

**Figure 5:**
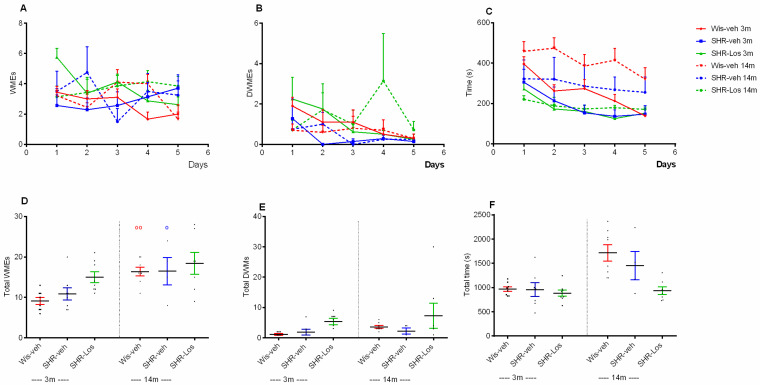
Effect of 3-month-old vehicle-treated Wistar rats (Wis-veh), vehicle-treated SHRs (SHR-veh), and losartan-treated SHRs (SHR-Los); and 14-month-old vehicle-treated Wistar rats (Wis-veh), vehicle-treated SHRs (SHR-veh), and losartan-treated SHRs (SHR-Los) on working memory errors (WMEs) (**A**), double working memory errors (DWMEs) (**B**), time to fulfill the task (**C**), total WMEs (**D**), total DWMEs (**E**), and total time over 5 sessions (**F**) measured in the radial arm maze (RAM) test. Data are presented as the mean ± SEM, (n = 8). Three-way ANOVA showed a main session effect: [F4,69 = 5.182, *p* = 0.002] and treatment effect: [F2,74 = 4.156, *p* = 0.02] (**A**); three-way ANOVA showed a main session effect: [F4,69 = 5.181, *p* < 0.002] (**C**). Two-way ANOVA showed a main effect of age: [F1,31 = 3.692, *p* = 0.041] (**D**). Post hoc Bonferroni test: ^oo^ *p* < 0.01, Wis-veh 14m vs. Wis-veh 3m; ^o^ *p* < 0.05, SHR-veh 14m vs. SHR-veh 3m. The color of the symbols corresponds to the color of the group with a significant difference.

The radial arm maze (RAM) test, introduced by Olton et al. (1976) [19], was used to measure both the ability of hungry rats to learn to find food using both types of information and the working memory for hippocampus-dependent spatial information (number of working memory errors and double working memory errors). While vehicle-treated Wistar rats (3m and 14m) learned the task over the 5-day session (*p* < 0.05, 5th session day vs. 1st session day), both young adult and middle-aged vehicle-treated SHRs did not improve their performance in the RAM test (*p* > 0.05, 5th session day vs. 1st session day) (Figure 5A–C). Both Wistar and vehicle-treated SHRs showed an age-related increase in total working memory errors (WMEs) (*p* < 0.01, Wis-veh 14m vs. 3m; *p* < 0.05, SHR-veh 14m vs. 3m) (Figure 5D). Overall, hippocampus-dependent spatial memory tested in the RAM was not affected by treatment factor (*p* > 0.05, SHR-Los vs. SHR-veh) (Figure 5D–F).

cAMP response element-binding protein (CREB) is a cellular transcription factor that plays a critical role in several physiological processes, including synaptic plasticity [20]. Its activation promotes the transcription of genes required for synaptic strengthening and memory storage. 

**Figure 6:**
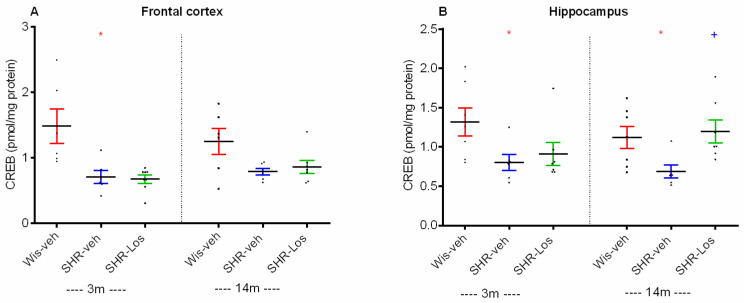
Effect of 3-month-old vehicle-treated Wistar rats (Wis-veh), vehicle-treated SHRs (SHR-veh), and losartan-treated SHRs (SHR-Los); and 14-month-old vehicle-treated Wistar rats (Wis-veh), vehicle-treated SHRs (SHR-veh), and losartan-treated SHRs (SHR-Los) on CREB expression in the frontal cortex (**A**) and hippocampus (**B**) measured by ELISA. Data are presented as mean ± SEM, (n = 6–7). Two-way ANOVA showed a main strain effect: [F1,25 = 12.792, *p* = 0.002] (**A**) and [F1,25 = 11.921, *p* = 0.002] (**B**); treatment effect: [F1,25 = 5.956, *p* = 0.023]. Post hoc Bonferroni test: * *p* < 0.05, SHR-veh 3m vs. Wis-veh 3m (**A**,**B**); * *p* < 0.05, SHR-veh 14m vs. Wis-veh 14m (**B**); ^+^ *p* < 0.05, SHR-Los 14m vs. SHR-veh 14m group (**B**). The color of the symbols corresponds to the color of the group with a significant difference.

Young adult and middle-aged vehicle-treated SHRs had decreased expression of CREB in the frontal cortex and the hippocampus compared to the age-matched groups of Wistar rats (*p* < 0.05, SHR-veh 3m vs. Wis-veh 3m; SHR-veh 14m vs. Wis-veh 14m) (Figure 6A,B). Aging did not affect the CREB expression of vehicle-treated SHRs either in the frontal cortex (FC) or the hippocampus (SHR-veh 14m vs. veh 3m) (*p* > 0.05) (Figure 6A,B). Long-term losartan treatment significantly increased the expression of CREB in the hippocampus of middle-aged SHRs compared with the vehicle-treated SHRs (*p* < 0.05) (Figure 6B).

**Figure 7:**
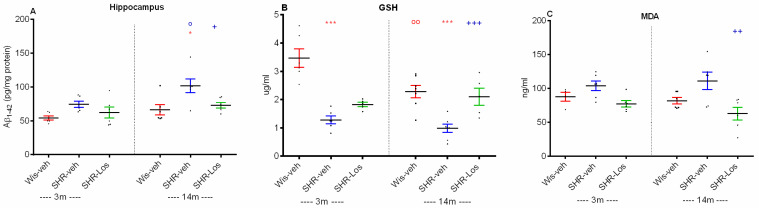
Effect of 3-month-old vehicle-treated Wistar rats (Wis-veh), vehicle-treated SHRs (SHR-veh), and losartan-treated SHRs (SHR-Los); and 14-month-old vehicle-treated Wistar rats (Wis-veh), vehicle-treated SHRs (SHR-veh), and losartan-treated SHRs (SHR-Los) on Aβ1-42 expression (**A**) and oxidative stress assessed by glutathione (GSH) level (**B**) and malondialdehyde (MDA) level (**C**) measured by ELISA test in the hippocampus. Data are presented as mean ± SEM, (n = 6–7). Two-way ANOVA showed a main effect of age: [F1,25 = 7.993, *p* = 0.01], strain: [F1,25 = 15.997, *p* < 0.001] (**A**); age: [F1,25 = 11.905, *p* = 0.002], strain: [F1,25 = 65.644, *p* < 0.001], treatment: [F1,25 = 21.732, *p* < 0.001] (**B**); atrain: [F1,25 = 8.118, *p* = 0.01], treatment: [F1,25 = 16.836, *p* < 0.001] (**C**). Post hoc Bonferroni test: * *p* < 0.05, SHR-veh 14m vs. Wis-veh 14m; ^o^ *p* < 0.05 SHR-veh 14m vs. SHR-veh 3m; ^+^ *p* < 0.05, SHR-Los 14m vs. SHR-veh 14m (**A**); ^oo^ *p* < 0.01, Wis-veh 14m vs. Wis-veh 3m; *** *p* < 0.001, SHR-veh 3m vs. Wis-veh 3m; *** *p* < 0.001, SHR-veh 14m vs. Wis-veh 14m; ^+++^ *p* < 0.001, SHR-Los 14m vs. SHR-veh 14m (**B**); ^++^ *p* < 0.01, SHR-Los 14m vs. SHR-veh 14m (**C**). The color of the symbols corresponds to the color of the group with a significant difference.

Middle-aged vehicle-treated SHRs showed higher Aβ_1-42_ expression in the hippocampus (*p* < 0.01) compared with the matched Wistar rats (*p* < 0.05, SHR-veh 14m vs. Wis-veh 14m) and young adult SHRs (*p* < 0.05, SHR-veh 14m vs. SHR-veh 3m) (Figure 7A). Losartan had a beneficial effect and reduced the age-related increase in Aβ_1–42_ in the hippocampus compared to the SHR-veh 14m group (*p* < 0.05) (Figure 7A). 

Glutathione (GSH) serves as a crucial antioxidant and detoxifying agent, while malondialdehyde (MDA) is a marker of lipid peroxidation and oxidative stress. Age-related decreases in GSH levels and increased MDA production are indicative of high oxidative stress during aging [21]. The role of aging and losartan treatment on oxidative stress in the hippocampus was evaluated by GSH and MDA levels.

Young adult and middle-aged vehicle-treated SHRs had reduced hippocampal GSH levels compared with the age-matched groups of Wistar rats (*p* < 0.001, SHR-veh 3m vs. Wis-veh 3m; SHR-veh 14m vs. Wis-veh 14m) (Figure 7B). Middle-aged Wistar rats also exhibited low levels of GSH compared with the young adult Wistar rats (*p* < 0.01, Wis-veh 14m vs. Wis-veh 3m). Long-term treatment with losartan increased the level of the endogenous antioxidant GSH in middle-aged rats compared with age-matched vehicle-treated SHRs (*p* < 0.001). Lipid peroxidation, as detected by the hippocampal MDA levels, was not significantly different in young adult and middle-aged Wistar rats and SHRs (Figure 7C). However, the lipid peroxidation was lower in middle-aged losartan-treated SHRs compared with vehicle-treated rats of the same age (*p* < 0.01) (Figure 7C).

## 3. Discussion

The main findings of the present study were that the antihypertensive drug losartan improved short-term recognition and spatial memory in SHRs in an age-dependent manner. Specifically, this antihypertensive drug was effective in middle-aged but not in young adult rats. Losartan treatment attenuated oxidative stress levels in middle-aged SHRs, suggesting a neuroprotective effect against age-related oxidative damage. In addition, the antihypertensive drug had a beneficial effect on critical biochemical markers related to memory and aging in the hippocampus such as CREB and the neurotoxic Aβ_1–42_ in middle-aged SHRs.

High blood pressure can increase the risk of Alzheimer’s disease (AD), the most common form of dementia [22]. It can increase the accumulation of amyloid plaques and tau tangles, the hallmarks of AD. Hypertension may impair various aspects of cognitive function, including memory, attention, processing speed, and executive function [1,23]. Cerebral dysfunction manifesting as dementia may be caused by untreated hypertension and associated vascular damage [22]. In particular, cerebrovascular pathology associated with an imbalance between endogenous antioxidants and oxidative stress and apoptosis predisposes to hypertension [24]. Due to similarities between hypertension-associated brain damage in humans and reported brain pathology such as neuronal loss, brain shrinkage, and glial activation [1,25], SHR is the most commonly used animal model to study the effects of hypertension on the brain. Johnson et al. (2020) [26] recently suggested that hypertension-induced microvascular dysfunction is an important mechanism underlying the hippocampus-dependent memory decline in SHRs. In the current study, young adult and middle-aged rats showed short-term recognition and spatial memory impairment. Similarly Hernandez et al. (2003) [9] reported that both young adult and middle-aged SHRs showed impaired cognitive performance. In addition, vehicle-treated middle-aged SHRs had lower SAB, suggesting impaired working memory. Although there was an age-dependent increase in systolic blood pressure in SHRs, spatial working memory tested in RAM did not differ between young adult SHRs and middle-aged SHRs [9]. In contrast to hypertensive rats, aging is a prerequisite for memory decline in normotensive strains, associated with altered neurotransmission, acetylcholinesterase activity, or an impaired antioxidant system [27,28,29,30]. Performance in the Morris water maze of older 18-month-old SHRs on the acquisition trials was also not significantly altered compared to younger age groups [31]. Our results are consistent with this finding, suggesting that the reduced learning ability of SHRs in the RAM test is not explicitly related to the age-related worsening of hypertension. Both hypertension and aging exacerbate brain damage and brain oxidative stress in the hippocampus of SHRs [10,32]. This exacerbation has been associated with increased apoptosis and astrocyte activation concomitant with reduced expression of PPAR-γ, which may contribute to the age-related brain damage observed in SHRs [10]. However, Yang et al. (2023) [33] provided evidence using the longitudinal fMRI method that the pathology of brain activity in SHRs is dependent on both hypertension and aging but there is no comorbidity between the two events. The reported increased expression of the neurotoxic oligomer Aβ_1–42_ in the hippocampus, but not in the FC, of middle-aged rats compared with young adult SHRs in the present study could be attributed to the blood’s origin due to the disrupted blood–brain barrier, as was reported by Wang et al. (2018) [34]. These authors showed that Aβ did not accumulate in neurons in the hippocampus and cortex of aged SHRs. The small group size in this work was a limitation, which requires further investigation focused on determining the origin of Aβ_1–42_ in the hippocampus.

In addition to being a model of hypertension, SHRs are also accepted as a valid model of attention deficit hyperactivity disorder (ADHD) [35], which is characterized by a severe working memory deficit [35]. Losartan treatment in SHRs improved short-term spatial and recognition memory (Y maze second test and ORT) but was unable to correct working memory as measured by the Y maze first test and the RAM test. Interestingly, unlike angiotensin-converting enzyme inhibitors (ACEIs), AT1 receptor blockers (ARBs), including losartan, were not only unable to prevent hyperlocomotion and impulsive-like behavior, but also exacerbated locomotor activity in “knockout” mice (NK1R-/-) with behavior resembling ADHD [36].

Overactivation of the renin–angiotensin system (RAS) plays a critical role in the aging process and the age-related pathogenesis of many diseases, including hypertension and AD. Interventions targeting this system, such as ACEIs and ARBs, have been reported to have potential benefits in mitigating the excessive cellular oxidation and impairment associated with this condition [37,38]. Evidence from the literature suggests that blocking the RAS may have pleiotropic age-protecting effects in a variety of pathological conditions in both animals and humans. For example, several studies have demonstrated the beneficial effects of treatment with RAS inhibitors, ACEIs, and ARBs, including losartan, on the pathogenesis associated with comorbid hypertension and mild dementia or the early form of AD in both humans and hypertensive rats [34,38,39]. Losartan treatment has been reported to have potent antioxidant effects and to protect against mitochondrial dysfunction in SHRs [40] and streptozotocin-induced diabetic rats [41]. The present study confirmed these findings for the antioxidant activity of losartan treatment in middle-aged rats but not in young adult rats. The drug dose used in our study was adjusted to produce an antihypertensive effect in SHRs and was similar to that used by other authors [42]. Under certain conditions such as hypertension, diabetes, and aging, there is an increased production of oxidants (reactive oxygen species) due to overactivation of the RAS, which can lead to tissue damage. The ACEI enalapril, by suppressing the synthesis of the pro-oxidant angiotensin II (Ang II), exerts a putative mitochondrial protection and potent antioxidant activity, thereby delaying the natural aging mechanisms [43]. The plausible explanation for the efficacy of losartan in middle-aged but not in young adult SHRs in the present study may be related to its beneficial effect on the hippocampal vasculature, which is thought to be more impaired in older rats, thereby contributing to its cognitive-improving effect. This effect may be related to the ability of losartan to reduce the expression of the oligomeric neurotoxin Aβ_1–42_ in the hippocampus in older SHRs but not in younger rats. Treatment with ARBs has been shown to reduce AD-associated pathology, and particularly, Aβ_1–42_, as assessed in the postmortem brains of patients [37]. Interestingly, other antihypertensive drugs have been shown to have no effect on brain amyloid burden, as recently confirmed by Deng et al. (2022) [40], who reported that patients with comorbid hypertension and a mild form of dementia showed promising improvement after treatment with ARBs compared with ACE inhibitors and other classes of antihypertensive drugs. Taken together, our results and the literature data suggest that the beneficial effects of losartan on comorbid hypertension and cognitive decline are age-dependent and are partly related to its potential to reduce oxidative stress burden in the hippocampus. An imbalance between endogenous antioxidants and pro-oxidants is thought to be a critical factor in AD-associated pathology such as cognitive decline caused by Aβ_1–42_ accumulation. The relationship between oxidative stress and Aβ_1–42_ is bidirectional and complex. However, oxidative damage often precedes the onset of clinical symptoms and the characteristic neuropathological features of AD, including Aβ deposition and vascular dysfunction [44]. These factors may exacerbate oxidative damage and create a cycle of neurodegeneration and cognitive decline. Therefore, our findings help to understand the mechanisms underlying the beneficial effect of losartan treatment on cognitive impairment in middle-aged SHRs, which may hold promise for delaying the onset or predisposition to the development of AD-type dementia associated with hypertension-related brain vascular damage. However, further research is needed to fully elucidate the complex interplay between oxidative stress and other pathological mechanisms in AD.

## 4. Materials and Methods

### 4.1. Animals and Experimental Design

The experiments in the present study were performed in full compliance with the European Communities Council Directive 2010/63/E.U. for animal experiments. In addition, the research project was approved by the Bulgarian Food Safety Agency (#300/N°5888–0183). Male Wistar rats and spontaneously hypertensive rats (SHRs) purchased from the Institute of Neurobiology, BAS vivarium, were used in this study at two ages: 3 months and 14 months. They were housed under standard environmental conditions. These included a constant 12 h light/12 h dark cycle, an average temperature maintained at 21 °C, and a humidity level of 45%. They were housed in groups of 3–4 rats in clear plastic cages of varying sizes depending on their age. The rats had free access to food and water throughout the study, except during testing periods. Six experimental groups were used as follows (n = 6–8): 3-month-old rats, (1) vehicle-treated Wistar rats (Wis-veh 3m), (2) vehicle-treated SHRs (SHR-veh) and (3) losartan-treated SHRs (SHR-Los 3m); and 14-month-old rats, (1) Wis-veh 14m, (2) SHR-veh 14m, and (3) SHR-Los 14m. Losartan was administered intraperitoneally (i.p.) at a dose of 10 mg/kg b.w. for 19 days. The matched control groups were injected with a vehicle (saline).

### 4.2. Behavioral Tests

#### 4.2.1. Measurement of Motor Activity

Motor activity and circadian oscillations were recorded using an actimeter (Bioseb, Paris, France). The analysis was performed as described in our previous study [45]. The following parameters were recorded: total distance and motor activity registered for 24 h.

#### 4.2.2. Memory Tests

##### Y-Maze Test

The Y-maze 1st test was used to assess working memory. Each rat was placed in the center of the apparatus and given free access to explore the three arms for 8 min. Access to the arms was manually recorded by two persons blind to the experimental procedure. We scored spontaneous alternation based on visits to three different arms, referred to as a triad. To calculate the SAB, we considered both the total number of arm visits and the number of triads. The SAB was calculated using the following formula:

SAB: % = (Total entries (N) − 2 × Number of correct entries × 100)(1)

In the second test (Y-maze 2nd test), both the time spent in and the number of entries into the familiar and the novel arms during this phase of the experiment were measured. The discrimination index was calculated using the following formula:

DI = (N)/(N + F)(2)

Between each test, the arms of the maze were wiped with alcohol to eliminate odors. 

##### Object Recognition Test (ORT)

The object recognition test (ORT) was performed according to the methodology described in our previous study [45]. Briefly, 24 h after the rats were habituated to an empty open-field (OF) apparatus measuring 50 × 50 × 50 cm^3^, they were placed in it next to two identical plastic objects (referred to as “F”) for 5 min (training phase). Sixty minutes later, the rats were again placed in the same box. However, during this test phase, one of the objects was replaced by a novel object (referred to as “N”). This phase also lasted for 5 min. During both the training and test phases, we carefully observed and recorded the time and number of times each object was explored by sniffing. The exploration time was measured in seconds and the count (number of sniffs) was recorded for each object. The discrimination index was calculated according to Formula (2). 

##### Radial Arm Maze (RAM)

The stainless steel RAM apparatus had an octagonal platform, with a 30 cm diameter, at its center. Each of the eight arms of the apparatus was identical, closed at the ends (12 cm × 12 cm), and positioned 50 cm above the floor. One week before the start of the test the animals were placed on a diet to reduce their body weight by 20%. The pretest (shaping) was performed for up to 15 min on three days. The rats were placed in a dimly lit room 30 min before the test. Test (up to 10 min): a pellet was placed at the end of each arm. The rat was placed in the center of the platform and allowed to freely search for available food. As consumed pellets were not replaced, each rat could enter each arm only once. The test ended when the rat had consumed all the pellets. The test was performed in five sessions, one per day. The following parameters were recorded: total WMEs, DWMEs, and time for five sessions. WME (number of working memory errors): The number of entries into an empty arm (with a consumed pellet) for the first time from day 1 to day 5 of the recording. DWME (number of double WMEs): The number of consecutive entries into an empty arm from day 1 to day 5 of the study. Time to consume all pellets: the time taken by each rat to consume all pellets during the test sessions.

### 4.3. Biochemistry

Three days after the last session in the RAM test, the rats were euthanized by decapitation after CO_2_ anesthesia. Left and right hippocampal and frontal cortex tissues were isolated and frozen in liquid nitrogen and stored in a fridge at −32 °C. The tissue samples were weighted and homogenized with HEPES buffer (20 mM HEPES; 1 mM EGTA; 210 mM mannitol; 70 mM sucrose; pH 7.2) and protease inhibition cocktail (100 mM PMSF, 100 mM NaF, 35 mM EDTA). The homogenates were centrifuged at 10,000× *g* for 5 min at 4 °C. The supernatants were used for determination of general protein via the Bradford method and afterward enzyme-linked immunosorbent assay (ELISA) tests were performed to determine oxidative stress markers of MDA (Elabscience E-EL-0060) and GSH (Elabscience E-EL-0026) in the hippocampus, along with Aβ (Elabscience E-EL-R1402) and CREB1 (Elabscience E-EL-R0289) in both hippocampal tissue and frontal cortex, according to the manufacturer’s protocol.

### 4.4. Statistical Analysis

Experimental data are presented as mean ± S.E.M. Two-way ANOVA was applied to the data of motor activity registered by the actimeter, memory measured by Y-maze test, ORT, total WMEs, DWMEs, time measured by the RAM test, and biochemical data with factors: age and strain (groups: Wis-veh 3m, Wis-veh 14m, SHR-veh 3, and SHR-veh 14m) and age and treatment (groups: SHR-veh 3m, SHR-Los 3m, SHR-veh 14m, and SHR-Los 14m). A three-way ANOVA was used for the RAM test, with strain and age as the between-subject factors and session day as the within-subject factor (groups: Wis-veh 3m, Wis-veh 14m, SHR-veh 3, and SHR-veh 14m), and age, treatment, and session day (groups: SHR-veh 3m, SHR-Los 3m, SHR-veh 14m, and SHR-Los 14m). When a significant difference was found, the ANOVA was followed by the Bonferroni post hoc test. Statistical analysis was performed with SigmaStat^®^ 11.0 (Systat Software, San Jose, CA, USA) and GraphPad Prism^®^6 (GraphPad Software, San Diego, CA, USA). Significant difference was accepted at *p* ≤ 0.05.

## 5. Conclusions

In conclusion, our study showed that young adult and middle-aged SHRs have impaired short-term recognition and spatial memory compared with age-matched Wistar rats. The results suggest that losartan is effective in improving cognitive performance in middle-aged SHRs but not in young adult rats. This suggests that the memory-improving effects of this selective AT1 receptor antagonist may not be solely due to its blood pressure-lowering properties but rather to its potent antioxidant activity. In addition, the study suggests that the protective effect of losartan in reducing Aβ_1–42_ expression in middle-aged rats may be closely related to the CREB mechanism in the hippocampus, further highlighting its potential neuroprotective and cognitive enhancement by mechanisms beyond its traditional antihypertensive function. Further research, particularly in aged SHR models and exploration of other AT_1_ receptor antagonists, is warranted to validate the hypothesis that the memory-enhancing effects of this class of antihypertensive drugs may be age-dependent.

## Data Availability

The data that support the findings of this study are available from the corresponding author upon reasonable request.

## References

[1] Feng R., Rolls E.T., Cheng W., Feng J. (2020). Hypertension Is Associated with Reduced Hippocampal Connectivity and Impaired Memory. EBioMedicine.

[2] Tucsek Z., Noa Valcarcel-Ares M., Tarantini S., Yabluchanskiy A., Fülöp G., Gautam T., Orock A., Csiszar A., Deak F., Ungvari Z. (2017). Hypertension-Induced Synapse Loss and Impairment in Synaptic Plasticity in the Mouse Hippocampus Mimics the Aging Phenotype: Implications for the Pathogenesis of Vascular Cognitive Impairment. GeroScience.

[3] Okamoto K., Aoki K. (1963). Development of a Strain of Spontaneously Hypertensive Rats. Jpn. Circ. J..

[4] Knardahl S., Karlsen K. (1984). Passive-Avoidance Behavior of Spontaneously Hypertensive Rats. Behav. Neural Biol..

[5] Sabbatini M., Strocchi P., Vitaioli L., Amenta F. (2000). The Hippocampus in Spontaneously Hypertensive Rats: A Quantitative Microanatomical Study. Neuroscience.

[6] Ferguson S.A., Cada A.M. (2003). A Longitudinal Study of Short- and Long-Term Activity Levels in Male and Female Spontaneously Hypertensive, Wistar-Kyoto, and Sprague-Dawley Rats. Behav. Neurosci..

[7] Nakamura-Palacios E.M., Caldas C.K., Fiorini A., Chagas K.D., Chagas K.N., Vasquez E.C. (1996). Deficits of Spatial Learning and Working Memory in Spontaneously Hypertensive Rats. Behav. Brain Res..

[8] Tchekalarova J., Krushovlieva D., Ivanova P., Kortenska L. (2023). Spontaneously Hypertensive Rats vs. Wistar Kyoto and Wistar Rats: An Assessment of Anxiety, Motor Activity, Memory Performance, and Seizure Susceptibility. Physiol. Behav..

[9] Hernandez C.M., Høifødt H. (2003). Spontaneously Hypertensive Rats: Further Evaluation of Age-Related Memory Performance and Cholinergic Marker Expression. J. Psychiatry Neurosci..

[10] Li Y., Liu J., Gao D., Wei J., Yuan H., Niu X., Zhang Q. (2016). Age-Related Changes in Hypertensive Brain Damage in the Hippocampi of Spontaneously Hypertensive Rats. Mol. Med. Rep..

[11] Linz W., Jessen T., Becker R.H.A., Schölkens B.A., Wiemer G. (1997). Long-Term ACE Inhibition Doubles Lifespan of Hypertensive Rats. Circulation.

[12] Linz W., Wohlfart P., Schoelkens B.A., Becker R.H.A., Malinski T., Wiemer G. (1999). Late Treatment With Ramipril Increases Survival in Old Spontaneously Hypertensive Rats. Hypertension.

[13] Chan S.-L., Bishop N., Li Z., Cipolla M.J. (2018). Inhibition of PAI (Plasminogen Activator Inhibitor)-1 Improves Brain Collateral Perfusion and Injury After Acute Ischemic Stroke in Aged Hypertensive Rats. Stroke.

[14] Benigni A., Cassis P., Remuzzi G. (2010). Angiotensin II Revisited: New Roles in Inflammation, Immunology and Aging. EMBO Mol. Med..

[15] Duchemin S., Belanger E., Wu R., Ferland G., Girouard H. (2013). Chronic Perfusion of Angiotensin II Causes Cognitive Dysfunctions and Anxiety in Mice. Physiol. Behav..

[16] Ongali B., Nicolakakis N., Tong X.-K., Aboulkassim T., Papadopoulos P., Rosa-Neto P., Lecrux C., Imboden H., Hamel E. (2014). Angiotensin II Type 1 Receptor Blocker Losartan Prevents and Rescues Cerebrovascular, Neuropathological and Cognitive Deficits in an Alzheimer’s Disease Model. Neurobiol. Dis..

[17] El-Mas M.M., Abdel-Rahman A.A. (2005). Longitudinal Studies on the Effect of Hypertension on Circadian Hemodynamic and Autonomic Rhythms in Telemetered Rats. Life Sci..

[18] Oosting J., Struijker-Boudier H.A., Janssen B.J. (1997). Circadian and Ultradian Control of Cardiac Output in Spontaneous Hypertension in Rats. Am. J. Physiol.-Heart Circ. Physiol..

[19] Olton D.S., Samuelson R.J. (1976). Remembrance of Places Passed: Spatial Memory in Rats. J. Exp. Psychol. Anim. Behav. Process..

[20] Lonze B.E., Ginty D.D. (2002). Function and Regulation of CREB Family Transcription Factors in the Nervous System. Neuron.

[21] Liguori I., Russo G., Curcio F., Bulli G., Aran L., Della-Morte D., Gargiulo G., Testa G., Cacciatore F., Bonaduce D. (2018). Oxidative Stress, Aging, and Diseases. CIA.

[22] Sáiz-Vazquez O., Puente-Martínez A., Pacheco-Bonrostro J., Ubillos-Landa S. (2023). Blood Pressure and Alzheimer’s Disease: A Review of Meta-Analysis. Front. Neurol..

[23] Kilander L., Nyman H., Boberg M., Hansson L., Lithell H. (1998). Hypertension Is Related to Cognitive Impairment: A 20-Year Follow-up of 999 Men. Hypertension.

[24] Harrison D.G., Gongora M.C. (2009). Oxidative Stress and Hypertension. Med. Clin. N. Am..

[25] Pietranera L., Saravia F., Gonzalez Deniselle M.C., Roig P., Lima A., De Nicola A.F. (2006). Abnormalities of the Hippocampus Are Similar in Deoxycorticosterone Acetate-Salt Hypertensive Rats and Spontaneously Hypertensive Rats. J. Neuroendocrinol..

[26] Johnson A.C., Miller J.E., Cipolla M.J. (2020). Memory Impairment in Spontaneously Hypertensive Rats Is Associated with Hippocampal Hypoperfusion and Hippocampal Vascular Dysfunction. J. Cereb. Blood Flow Metab..

[27] Foster T.C. (2012). Dissecting the Age-Related Decline on Spatial Learning and Memory Tasks in Rodent Models: N-Methyl-D-Aspartate Receptors and Voltage-Dependent Ca^2+^ Channels in Senescent Synaptic Plasticity. Prog. Neurobiol..

[28] Gerrard J.L., Burke S.N., McNaughton B.L., Barnes C.A. (2008). Sequence Reactivation in the Hippocampus Is Impaired in Aged Rats. J. Neurosci..

[29] Scali C., Giovannini M.G., Prosperi C., Bartolini L., Pepeu G. (1997). Tacrine Administration Enhances Extracellular Acetylcholinein Vivoand Restores the Cognitive Impairment in Aged Rats. Pharmacol. Res..

[30] Haider S., Saleem S., Perveen T., Tabassum S., Batool Z., Sadir S., Liaquat L., Madiha S. (2014). Age-Related Learning and Memory Deficits in Rats: Role of Altered Brain Neurotransmitters, Acetylcholinesterase Activity and Changes in Antioxidant Defense System. AGE.

[31] Skinner M.H., Tan D.-X., Grossmann M., Pyne M.T., Mahurin R.K. (1996). Effects of Captopril and Propranolol on Cognitive Function and Cerebral Blood Flow in Aged Hypertensive Rats. J. Gerontol. Ser. A Biol. Sci. Med. Sci..

[32] Lee M.-C., Shoji H., Miyazaki H., Yoshino F., Hori N., Toyoda M., Ikeda Y., Anzai K., Ikota N., Ozawa T. (2004). Assessment of Oxidative Stress in the Spontaneously Hypertensive Rat Brain Using Electron Spin Resonance (ESR) Imaging and in Vivo L-Band ESR. Hypertens. Res..

[33] Yang Y., Zhu Q., Wang L., Gao D., Wang Z., Geng Z. (2023). Effects of Hypertension and Aging on Brain Function in Spontaneously Hypertensive Rats: A Longitudinal Resting-State Functional Magnetic Resonance Imaging Study. Cereb. Cortex.

[34] Wang Y., Zhang R., Tao C., Xu Z., Chen W., Wang C., Song L., Zheng J., Gao F. (2018). Blood-Brain Barrier Disruption and Perivascular Beta-Amyloid Accumulation in the Brain of Aged Rats with Spontaneous Hypertension: Evaluation with Dynamic Contrast-Enhanced Magnetic Resonance Imaging. Korean J. Radiol..

[35] Kantak K.M., Singh T., Kerstetter K.A., Dembro K.A., Mutebi M.M., Harvey R.C., Deschepper C.F., Dwoskin L.P. (2008). Advancing the Spontaneous Hypertensive Rat Model of Attention Deficit/Hyperactivity Disorder. Behav. Neurosci..

[36] Kofler M.J., Singh L.J., Soto E.F., Chan E.S.M., Miller C.E., Harmon S.L., Spiegel J.A. (2020). Working Memory and Short-Term Memory Deficits in ADHD: A Bifactor Modeling Approach. Neuropsychology.

[37] De Cavanagh E.M.V., Inserra F., Ferder M., Ferder L. (2007). From Mitochondria to Disease: Role of the Renin-Angiotensin System. Am. J. Nephrol..

[38] Santos C.R.D., Grigorova Y.N., McDevitt R.A., Long J.M., Cezayirli D., Zernetkina V., Wei W., Haghkar M., Morrell C.H., Juhasz O. (2022). Treatment with Losartan, an AT1 Receptor Blocker, Improves Cognitive and Cardiovascular Function in a Dahl Salt-sensitive Rat Model of Age-associated Vascular Dementia. Alzheimer’s Dement..

[39] Hajjar I., Brown L., Mack W.J., Chui H. (2012). Impact of Angiotensin Receptor Blockers on Alzheimer Disease Neuropathology in a Large Brain Autopsy Series. Arch. Neurol..

[40] Deng Z., Jiang J., Wang J., Pan D., Zhu Y., Li H., Zhang X., Liu X., Xu Y., Li Y. (2022). Angiotensin Receptor Blockers Are Associated With a Lower Risk of Progression From Mild Cognitive Impairment to Dementia. Hypertension.

[41] Kamper M., Tsimpoukidi O., Chatzigeorgiou A., Lymberi M., Kamper E.F. (2010). The Antioxidant Effect of Angiotensin II Receptor Blocker, Losartan, in Streptozotocin-Induced Diabetic Rats. Transl. Res..

[42] Khattab M., Ahmad M., Al-Shabanah O.A., Raza M. (2004). Effects of Losartan on Blood Pressure, Oxidative Stress, and Nitrate/Nitrite Levels in the Nitric Oxide Deficient Hypertensive Rats. Recept. Channels.

[43] De Cavanagh E.M.V., Inserra F., Ferder L. (2011). Angiotensin II Blockade: A Strategy to Slow Ageing by Protecting Mitochondria?. Cardiovasc. Res..

[44] Tamagno E., Guglielmotto M., Vasciaveo V., Tabaton M. (2021). Oxidative Stress and Beta Amyloid in Alzheimer’s Disease. Which Comes First: The Chicken or the Egg?. Antioxidants.

[45] Tchekalarova J., Krushovlieva D., Ivanova P., Nenchovska Z., Toteva G., Atanasova M. (2024). The Role of Melatonin Deficiency Induced by Pinealectomy on Motor Activity and Anxiety Responses in Young Adult, Middle-Aged and Old Rats. Behav. Brain Funct..

